# *In vitro* Antibacterial Activity of *Combretum edwardsii, Combretum krausii*, and *Maytenus nemorosa* and Their Synergistic Effects in Combination with Antibiotics

**DOI:** 10.3389/fphar.2016.00208

**Published:** 2016-07-15

**Authors:** Jude C. Chukwujekwu, Johannes van Staden

**Affiliations:** Research Centre for Plant Growth and Development, School of Life Sciences, University of KwaZulu-NatalPietermaritzburg, South Africa

**Keywords:** antibacterial activity, synergistic effects, *Maytenus nemorosa*, *Combretum edwardsii*, *Combretum krausii*

## Abstract

The study investigated the antibacterial activity of crude extracts of *C. edwardsii, Combretum krausii*, and *Maytenus nemorosa* as well as their interactions with selected antibiotics against drug resistant bacterial strains. Using the rapid p-iodonitrotetrazolium chloride colorimetric assay, minimum inhibitory concentration values of plant extracts and antibiotics were determined. The interactions of plant extracts and antibiotics were studied using a checkerboard method. The MICs of the plant extracts and antibiotics were in the range of 0.037–6.25 and 0.001–2.5 mg/ml, respectively. The plant fractions tested in the present study displayed varying levels of antibacterial activity depending on the bacterial strains. Generally, *Staphylococcus aureus* was the most susceptible of the three strains of bacteria while the other two beta-lactamase producing Gram-negative bacteria were the most resistant. The hexane leaf extract of *M. nemorosa* was the most active (MIC = 37 μg/ml) against *S. aureus*. Ethyl acetate leaf extract of *C. krausii* was the most active against *Klebsiella pneumoniae* and ethyl acetate leaf extract of *C. edwardsii* was the most active against *Escherichia coli*. Synergistic interactions were detected in 13% of the combinations against *E. coli*, 27% of the combinations against *K. pneumoniae* and 80% of the combinations against *S. aureus*. The few synergistic interactions observed in the present study suggest that the crude extracts of the leaves of *M. nemorosa, C. edwardsii*, and *C. krausii* could be potential sources of broad spectrum antibiotic resistance modifying compounds.

## Introduction

The emergence and spread of drug-resistant bacteria remains a major challenge to public health in the treatment of bacterial infections. Due to this resistance, the clinical efficacy of current antimicrobial agents is decreasing against many pathogens. Some of the commonly used antibiotics, especially beta-lactam antibiotics are rendered infective through some resistance mechanisms employed by drug-resistant bacteria. One of such resistance mechanisms is the hydrolysis of the active site, beta lactam ring, of beta-lactam antibiotics by beta-lactamases, and thereby rendering the antibiotic ineffective. Beta-lactamases are classified into four different classes (A, B, C, and D) based on structural comparisons or four groups (1–4) based on hydrolytic and inhibitor profiles (Ambler et al., 1991; Bush and Jacoby, 2010). Class A, C, and D beta-lactamases use a serine as a nucleophile to hydrolyze the beta-lactam bond while class B beta-lactamases (carbapenemases) use Zn^2+^ to deactivate beta-lactams. Beta-lactamases are the most significant and prevalent mechanism of resistance to beta-lactams. Members of the family Enterobacteriaceae commonly express plasmid-encoded beta-lactamases (TEM and SHV) which confer resistance to penicillins but not to expanded-spectrum cephalosporins. TEM-1 (class A) is the most commonly encountered beta-lactamase in Gram-negative bacteria. Up to 90% of ampicillin resistance in *E. coli* is due to the production of TEM-1 (Cooksey et al., 1990) while the most common class A beta-lactamases found in *Klebsiella* are the chromosomal and plasmid-borne SHV enzymes and the plasmid-mediated TEM enzymes (Bush, 2001). Combination therapy has over the years become one of the most effective strategies in combating bacterial infections caused by drug resistant pathogens. The rationale is to enhance the activity by achievement of a synergistic effect. According to Rasoanaivo et al. (2011), “synergy” or “potentiation” means that the effect of the combination is greater than the sum of the individual effects. Synergistic effects manifest in different ways: improving bioavailability; decreasing metabolism, and excretion of the active component; reversal of resistance; and modulation of adverse effects (Wagner and Ulrich-Merzenich, 2009; Rasoanaivo et al., 2011). Combination therapy is becoming a theme of infectious diseases and is increasingly being accepted as a reducer of microbial resistance.

Medicinal plants are known to be very rich in phytochemicals with diverse biological activities. Researchers have shown that co-occurring compounds in medicinal plants play a role in enhancing the bioavailability and distribution of various phytochemicals, and reversal of resistance (Butterweck et al., 2003; Rath et al., 2004).

*Maytenus nemorosa* (Eckl. & Zeyh.) Marais [Syn: *Gymnosporia nemorosa* (Eckl. & Zeyh.) Szyszyl.], (Celastraceae), is a spiny evergreen shrub or small tree with drooping branches growing up to 5 m tall. It grows on forest edges in Mpumalanga, Swaziland, and KwaZulu-Natal, Eastern, and Western Cape. As far as we know, there is no information on the use of *M. nemorosa* in traditional medicine. However, some members of the genus, notably *Maytenus senegalensis* and *Maytenus acuminate* are used to treat a wide range of ailments such as pneumonia, tuberculosis, venereal diseases, epilepsy, diarrhea, sore throats and stomach ailments (Pooley, 1993).

The genus *Combretum*, a member of the plant family Combretaceae are found mainly in tropical and subtropical areas of Africa and Asia. Some members of the genus are used in traditional medicine to treat a wide range of ailments that include inflammation, infections, diabetes, malaria, bleeding and diarrhea. *Combretum krausii* Hochst, commonly known as the forest bush willow, is a medium-sized to large tree found in Eastern South Africa, Swaziland and Southern Mozambique (Van Wyk, 1997; Le Roux and Reynolds, 2003). It is used as local medicine as antiseptic, antidiuretic, tonic and appetite stimulant, to treat wounds, and eye infections (Quattrocchi, 2012). As far as we know there is no record of ethnopharmacological use of *Combretum edwardsii* Exell. Limited information exists on the biological activities of both *C. edwardsii* and *C. krausii*. Antiinflammatory and antioxidant activites were reported for both Combretum species (McGaw et al., 2001; Masoko and Eloff, 2007). A derivative of mollic acid; 1α-hydroxycycloartenoid mollic acid α-l-arabinoside was isolated from *C. edwardsii* (Rogers, 1989) while uteroactive compounds; combretastatin, allegic acid and their derivatives were reported for *C. krausii* (Brookes et al., 1999).

In line with the ethnobotanical use of *C. edwardsii, C. krausii, M. nemorosa*, the present work was designed to evaluate the antibacterial activities of *C. edwardsii, C. krausii, M. nemorosa* as well as their interactions with selected antibiotics against drug-resistant bacterial strains.

## Materials and methods

### Plant collection

The plant materials used in the present study were leaves of *M. nemorosa* (Eckl. & Zeyh.) Marais, *C. edwardsii* Exell. and *C. krausii* Hochst. They were collected in January, 2015 from the Botanical Garden of the University of KwaZulu-Natal, Pietermaritzburg, South Africa. The plants were appropriately identified by the Curator of the garden and voucher specimens (*C. edwardsii*—Chukwujekwu #8 NU; *C. krausii*—Chukwujekwu #9 NU; *M. nemorosa*—Chukwujekwu #10 NU) were deposited in the Herbarium of the University of KwaZulu-Natal, Pietermaritzburg.

### Preparation of plant extracts

Plant materials were dried at 50°C (in the dark), powdered and stored in paper containers at ambient temperature for < 24 h prior to extraction. The oven-dried powdered leaves (30 g of each plant) were extracted with 80% methanol (300 ml) with sonication for 1 h and then soaked overnight. The extracts were filtered through a Büchner funnel using Whatman No.1 filter paper, and the solvent evaporated under reduced pressure at 30°C. Liquid–liquid partitioning was done by dissolving the crude extracts (*M. nemorosa* = 954 mg; *C. edwardsii* = 1070 mg; *C. krausii* = 983 mg) in aqueous methanol (250 ml, 80% v/v) followed by extraction with hexane three times (3 × 300 ml) in a separating funnel. The hexane layers were combined and dried under reduced pressure, and the remaining aqueous layer was concentrated by evaporation of the methanol (MeOH) under reduced pressure and then diluted with distilled water to a volume of 300 ml. The aqueous residues were extracted three times with dichloromethane (3 × 300ml) in a separating funnel. The dichloromethane layers were combined and dried under reduced pressure. The aqueous layer was concentrated and diluted as described earlier and then extracted with ethyl acetate. Finally, the aqueous fraction was first concentrated under reduced pressure and subsequently freeze dried. The four fractions (hexane, dichloromethane, ethyl acetate, and aqueous) were used in the present study.

### Chemicals for antimicrobial assays

P-Iodonitrotetrazolium chloride (INT; Sigma-Aldrich) was used as microbial growth indicator and Chloramphenicol (CHL), Ampicillin (AMP), Amoxicillin (AMX), Penicillin (PEN), Cefotaxime (CEF; Sigma-Aldrich) were used as reference antibiotics.

### Preparation of microorganisms

The bacteria used in this study were *Klebsiella pneumoniae* ATCC 700603, *Escherichia coli* ATCC 25218, *Staphylococcus aureus* ATCC 11632. The cultures of bacteria were maintained on Mueller Hinton Agar (MHA) slants at 4°C throughout the study and used as stock cultures.

### Determination of minimum inhibitory concentration (MIC)

The MIC values of plant extracts and antibiotics against the bacterial strains were determined using a rapid p-iodonitrotetrazolium chloride (INT) colorimetric assay (Eloff, 1998). Stock solutions of plant extracts (100 mg/ml) and antibiotic (10 mg/ml) were prepared with absolute ethanol and sterilized distilled water, respectively. Prior to the assay, stock solutions (plant extracts) were subsequently diluted with sterilized distilled water to a concentration of 12.5 mg/ml. These (plant extracts and antibiotics) were then added to Mueller Hinton Broth (MHB), and serially diluted 2-fold in a 96-well-microplate to a final concentration range of 3120–24 μg/ml for plant extracts and 2500–0.61 μg/ml for antibiotics. Bacterial strains were cultured overnight at 37°C on MHB and adjusted to a final density of 10^6^ cfu/ml with MHB. These were subsequently used as inocula. One hundred microliters (100 μl) of inoculum was added to each well. The plates were covered with a sterile plate sealer and then incubated at 37°C for 20 h. Wells containing 20% aqueous ethanol, MHB and 100 μl of inoculum served as the negative controls. The total volume in each well was 200 μl. The MICs of samples were observed after 20 h incubation at 37°C, and subsequent 30 min incubation after the addition 40 μl of 0.2 mg/ml INT. Clear wells with INT after incubation indicate inhibition of bacterial growth. Minimum inhibitory concentration (MIC) values were recorded as the lowest concentration of the sample that completely inhibited bacterial growth.

### Determination of *In vitro* synergistic activity

Combinations of the plant extracts and antibiotics were tested by the checkerboard method. For each plant extract and antibiotics combination, fifty microliters of MHB was added in each well of a 96-well-microplate. Fifty microliters of each plant extract was added in row A and was 2-fold serially diluted down to row H. Fifty microliters of appropriate dilutions of antibiotics was added in columns with column one having the highest concentration and column eight the lowest concentration of antibiotics. To the 100 μl of different combinations in each of the 96-well-microplates, 100 μl of bacterial inoculum as described earlier, was added. The final concentration of plant extract and antibiotics in combinations ranged from 1/64 times the MIC (1/64 × MIC) to 2 × MIC. Further, dilutions were made where the lowest concentrations of the plant extracts and antibiotics in combination inhibited the growth of the test organism. Plates were incubated for 20 h. Interpretation of the data was achieved by calculating the fractional inhibitory concentration index (FICI) as follows:

FIC A + FIC B.
FIC A = (MIC of sample A in combination with antibiotics/MIC of sample A alone).
FIC B = (MIC of antibiotics A in combination with sample/MIC of antibiotics A alone).

The results were interpreted as follows: FICI ≤ 0.5, synergistic; 0.5 < FICI < 4, no interaction; FICI ≥ 4 antagonistic (Odds, 2003).

## Results and discussion

The MICs of the plant extract fractions and standard antibiotics are shown in Table 1. The MICs of the fractions and standard antibiotics were in the range of 37–6250 and 1–2500 μg/ml, respectively. The plant fractions tested in the present study displayed varying levels of antibacterial activity depending on the bacterial strains (Table 1). Generally, *S. aureus* was the most susceptible of the three strains of bacteria while the other two beta-lactamase producing Gram-negative bacteria were the most resistant. The antibacterial activity of a plant extract is considered significant when its MIC value is below 100 μg/ml, moderate when ≤ 625 μg/ml and weak when it is above 625 μg/ml (Rios and Recio, 2005; Kuete, 2010). All the fractions of *M. nemorosa*, except the water fraction, showed the highest activity against *S. aureus*, the hexane fraction being the most active. The antibacterial activities of hexane, dichloromethane and ethyl acetate fractions of *M. nemorosa* against *S. aureus* could be considered significant. No information on the antibacterial activity of *M. nemorosa* and its phytochemical constituents in the literature. However, many species of *Maytenus* are known for their antibacterial activities and antibacterial compounds have also been isolated and identified from many of the species (Matu and van Staden, 2003; Lindsey et al., 2006; De León et al., 2010). The antibacterial activities of the two *Combretum* species also vary depending on the test organism. With respect to the *Combretum* fractions, the dichloromethane fraction of *C. edwardsii* displayed the best antibacterial activity (MIC = 0.195 μg/ml) against *S. aureus* (Table 1) while its ethyl acetate fraction showed the best activity (MIC = 390 μg/ml) against *E. coli*. All the fractions of *C. edwardsii* displayed relatively weak activity against *K. pneumoniae* (Table 1) whereas those of *C. Krausii*, except the water fraction, showed moderate antibacterial activity against *S. aureus* and weak activity against *E. coli* and *K. pneumoniae* (Table 1). Little or no information exist on the antibacterial activity and phytochemical constituents of *C. edwardsii* and *C. krausii*. Nonetheless, members of the genus *Combretum* have been extensively investigated for their antibacterial activity (Elegami et al., 2002; Fyhrquist et al., 2002; Eloff et al., 2005; Fankam et al., 2015). Antibacterial compounds have been isolated and identified within this genus. Martini et al. (2004) isolated and characterized five antibacterial flavonoids from *C. erythrophyllum*. Other antibacterial phytochemicals from *Combretum* species include stilbenoids, and triterpenoids (Angeh et al., 2007; Katerere et al., 2012). This is the first report of antibacterial activity of *C. edwardsii*. *C. krausii* has previously been investigated for antibacterial activity (Eldeen et al., 2005), but not against drug-resistant bacteria. The MICs of the standard antibiotics vary. Following the trends of antimicrobial activity pattern displayed by the plant extracts fractions; the standard antibiotics were more active against *S. aureus* than *E. coli* and *K. pneumoniae*. The duo Gram-negative bacteria showed strong resistance to ampicillin, amoxicillin, and penicillin. The results showed the multi-drug resistant status of the two Gram-negative bacteria. They were less resistant to cefotaxime and chloramphenicol. Cefotaxime was the most active of all the standard antibiotics used in the present study. The weak antibacterial activities exhibited by the fractions and some of the antibiotics against the two Gram-negative bacteria could be due to the multidrug-resistant nature of the bacteria. These are Gram-negative drug resistant bacteria that exhibit different mechanisms of resistance to different antibiotics. Unlike Gram-positive bacteria, the peptidoglycan layer of Gram-negative bacteria is surrounded by a second membrane comprised of a bilayer of phospholipids and lipopolysaccharide known as the outer membrane. This provides an extra layer of protection for the cell as compared to Gram-positive bacteria. It plays a vital role in preventing the diffusion of many antibiotics into the cell thereby preventing the drugs from reaching their intercellular targets to confer antibiotic activity (Worthington and Melander, 2013). The Gram-negative bacteria used in the present study are also beta-lactamase producing bacteria. These enzymes are located in the bacterial cell wall and they play a significant role in conferring antibacterial resistance on the bacterial cells by hydrolyzing many classes of antibiotics especially the beta-lactams that target cell wall synthesis (Worthington and Melander, 2013). The penicillin, ampicillin, and amoxicillin used in the present study are all beta-lactam antibiotics and they inhibit the synthesis of bacterial cell wall. The very weak antibacterial activities of penicillin, ampicillin, and amoxicillin observed in the present study confirmed the multi-drug resistant profile of the two Gram-negative bacteria. The Gram-negative bacteria displayed more resistant to beta-lactam antibiotics than the rest of the antibiotics. Cefotaxime is a third generation of Cephalosporins which targets the synthesis of bacterial cell wall while chloramphenicol targets the synthesis of protein within the bacterial cell. They both displayed better antibacterial activity than ampicillin, amoxicillin, and penicillin. This could be attributed to the fact that they are not beta-lactam antibiotics and hence do not get hydrolyzed by beta-lactamases.

**Table 1:** **MICs of different fractions of *Combretum edwardsii* (CE), *Combretum krausii* (CK), and *Maytenus nemorosa* (MN) and antimicrobial agents against test organisms**.

**Antimicrobial agents and extracts**	**MIC (μg/ml)**		
	**E. coli**	**K. pneumonia**	**S. aureus**
Ampicillin (AMP)	2500	2500	39
Amoxicillin (AMX)	2500	2500	39
Cefotaxime (CEF)	4	63	1
Chloramphenicol (CHL)	63	63	16
Penicillin (PEN)	2500	2500	39
CEH	1560	1560	390
CED	3125	1560	195
CEE	390	1560	780
CEW	3125	3125	6250
CKH	780	1560	390
CKD	780	1560	390
CKE	1560	780	390
CKW	3125	1560	1560
MNH	1560	1560	37
MND	3125	1560	49
MNE	3125	3125	98
MNW	6250	3125	6250

*Values represent triplicates of two independent experiments.CEH, Hexane fraction of Combretum edwardsii; CED, Dichloromethane fraction of Combretum edwardsii; CEE, Ethyl acetate fraction of Combretum edwardsii; CEW, Water fraction of Combretum edwardsii; CKH, Hexane fraction of Combretum krausii; CKD, Dichloromethane fraction of Combretum krausii; CKE, Ethyl acetate fraction of Combretum krausii; CKW, Water fraction of Combretum krausii; MNH, Hexane fraction of Maytenus nemorosa; MND, Dichloromethane fraction of Maytenus nemorosa; MNE, Ethyl acetate fraction of Maytenus nemorosa; MNW, Water fraction of Maytenus nemorosa*.

Synergistic activities resulting from the combinations of plant extracts with antibiotics are well known in the literature (Hübsch et al., 2014; Sahu et al., 2014). In the present study, we explored the possible synergistic effects of combinations of fractions of *C. krausii, C. edwardsii*, and *M. nemorosa* with selected antibiotics, respectively, against drug resistant pathogenic bacterial strains. The MICs obtained by the combinations of leaf extract fractions of *C. edwardsii, C. krausii*, and *M. nemorosa* with ampicillin, amoxicillin, cefotaxime, chloramphenicol, and penicillin, respectively, against *E. coli, K. pneumonia*, and *S. aureus* are presented in Tables 2–**4**. Plant secondary metabolites are known to possess antimicrobial activity. However, when used in combination, they possess the potential to either inhibit the modified target or exhibit a synergy by blocking one or more of the targets in the metabolic pathway thus acting as a modifier of multidrug resistance mechanisms (Hemaiswarya et al., 2008). The *E. coli* used in the present study is a Tem-1 beta-lactamase-producing strain (Non-ESBL). There were four synergistic (FICI ≤ 0.5) activities detected out of 30 combinations against *E. coli* (Table 2). All the synergistic activities were combinations with cefotaxime. There were 8–16-fold decreases in the MIC of cefotaxime in all the synergistic activities detected against *E. coli*. Similarly, the MICs of hexane fraction of *C. edwardsii*, dichloromethane, and ethyl acetate fractions of *C. krausii* and hexane fraction of *M. nemorosa* in combinations with cefotaxime, respectively, that produced synergistic activities, were lowered between 1/130 and 1/260. The best synergistic interaction (FICI = 0.064) was the combination between ethyl acetate fraction of *C. krausii* leaf extract and cefotaxime, followed by hexane fraction of *C. edwardsii* and cefotaxime (0.07), dichloromethane fraction of *C. krausii* and cefotaxime (0.07), and hexane fraction of *M. nemorosa* and cefotaxime (0.19).

**Table 2:** **Combined effects of different fractions of *Combretum edwardsii, Combretum krausii, and Maytenus nemorosa* with antibiotics against drug-resistant *Escherichia coli***.

**Combinations**	**Individual MIC (μg/ml)**	**Combination MIC (μg/ml)**	**Individual FIC**	**FIC index (FICI)**	**Interpretation**
CEH + AMP	1560/2500	24/1250	0.02/0.5	0.52	No interaction
CEE + AMP	390/2500	12/1250	0.03/0.5	0.53	No interaction
CEH + AMX	1560/2500	24/1250	0.02/0.5	0.52	No interaction
CEE + AMX	390/2500	12/2500	0.03/1	1.03	No interaction
CEH + CEF	1560/4	12/0.25	0.01/0.06	0.07	Synergistic
CEE + CEF	390/4	3/4	0.01/1	1.01	No interaction
CEH + CHL	1560/63	24/125	0.02/1.98	2.00	No interaction
CEE + CHL	309/63	50/63	0.13/1	1.13	No interaction
CEH + PEN	1560/2500	12/2500	0.01/1	1.01	No interaction
CEE + PEN	390/2500	12/2500	0.03/1	1.03	No interaction
CKH + AMP	780/2500	98/1250	0.13/0.5	0.63	No interaction
CKD + AMP	780/2500	24/1250	0.03/0.5	0.53	No interaction
CKE + AMP	1560/2500	1560/157	1/0.06	1.06	No interaction
CKH + AMX	780/2500	390/1250	0.5/0.5	1	No interaction
CKD + AMX	780/2500	49/1250	0.06/0.5	0.56	No interaction
CKE + AMX	1560/2500	1560/625	1/0.25	1.25	No interaction
CKH + CEF	780/4	390/0.5	0.5/0.13	0.63	No interaction
CKD + CEF	780/4	6/0.25	0.01/0.06	0.07	Synergistic
CKE + CEF	1560/4	6/0.25	0.004/0.06	0.064	Synergistic
CKH + CHL	780/63	780/8	1/0.13	1.13	No interaction
CKD + CHL	780/63	24/63	0.03/1	1.03	No interaction
CKE + CHL	1560/63	49/63	0.03/1	1.03	No interaction
CKH + PEN	780/2500	12/2500	0.02/1	1.02	No interaction
CKD + PEN	780/2500	12/2500	0.02/1	1.02	No interaction
CKE + PEN	1560/2500	1560/156	1/0.06	1.06	No interaction
MNH + AMP	1560/2500	409/2500	0.03/1	1.03	No interaction
MNH + AMX	1560/2500	1560/1250	1/0.5	1.5	No interaction
MNH + CEF	1560/4	195/0.25	0.13/0.06	0.19	Synergistic
MNH + CHL	1560/63	1560/8	1/0.13	1.13	Synergistic
MNH + PEN	1560/2500	12/2500	0.01/1	1.01	Synergistic

*Values represent triplicates of two independent experiments.CEH, Hexane fraction of Combretum edwardsii; CEE, Ethyl acetate fraction of Combretum edwardsii; CKH, Hexane fraction of Combretum krausii; CKD, Dichloromethane fraction of Combretum krausii; CKE, Ethyl acetate fraction of Combretum krausii; MNH, Hexane fraction of Maytenus nemorosa*.

A multidrug resistant strain of *K. pneumoniae* was used in the present study. It is an extended-spectrum beta-lactamase strain that produces the enzyme SHV-18. Against *K. pneumoniae*, 12 synergistic effects were detected out of 45 combinations (Table 3). The combinations of plant extracts with cefotaxime produced synergistic effects. The best being the combinations of ethyl acetate fraction of *C. edwardsii* and cefotaxime (FICI = 0.03), hexane fraction of *C. edwardsii* and cefotaxime (FICI = 0.06), dichloromethane fraction of *C. krausii* and cefotaxime (FICI = 0.06), and hexane fraction of *C. krausii* and cefotaxime (FICI = 0.09). In the combination of ethyl acetate fraction of *C. edwardsii* and cefotaxime, the MICs of cefotaxime and the plant extract were lowered from 63 to 2 μg/ml (1/32 of MIC) and from 1560 to 3 μg/ml (1/520 of MIC), respectively. Synergistic effects were also observed in the combinations of chloramphenicol with hexane fraction of *M. nemorosa* (FICI = 0.14), dichloromethane fraction of *M. nemorosa* (FICI = 0.38), and hexane fraction of *C. krausii* (FICI = 0.38), respectively.

**Table 3:** **Combined effects of different fractions of *Combretum edwardsii, Combretum krausii, and Maytenus nemorosa* with antibiotics against multidrug resistant *Klebsiella pneumonia***.

**Combinations**	**Individual MIC (μg/ml)**	**Combination MIC (μg/ml)**	**Individual FIC**	**FIC index (FICI)**	**Interpretation**
CEH + AMP	1560/2500	781/157	0.5/0.06	0.56	No interaction
CED + AMP	1560/2500	49/2500	0.03/1	1.03	No interaction
CEE + AMP	1560/2500	1560/313	1/0.13	1.13	No interaction
CEH + AMX	1560/2500	24//2500	0.02/1	1.02	No interaction
CED + AMX	1560/2500	49/2500	0.03/1	1.03	No interaction
CEE + AMX	1560/2500	1560/313	1/0.13	1.13	No interaction
CEH + CEF	1560/63	3/4	0.002/0.06	0.06	Synergistic
CED + CEF	1560/63	49/8	0.03/0.12	0.15	Synergistic
CEE + CEF	1560/63	3/2	0.002/0.03	0.03	Synergistic
CEH + CHL	1560/63	24/63	0.02/1	1.02	No interaction
CED + CHL	1560/63	195/31	0.13/0.49	0.62	No interaction
CEE + CHL	1560/63	200/31	0.13/0.5	0.63	No interaction
CEH + PEN	1560/2500	12/5000	0.01/2	2.01	No interaction
CED + PEN	1560/2500	12/5000	0.01/2	2.01	No interaction
CEE + PEN	1560/2500	12/5000	0.03/2	2.03	No interaction
CKH + AMP	1560/2500	1560/157	1/0.06	1.06	No interaction
CKD + AMP	1560/2500	1560/157	1/0.06	1.06	No interaction
CKE + AMP	780/2500	0.78/157	1/0.03	1.03	No interaction
CKW + AMP	1560/2500	1560/157	1/0.06	1.06	No interaction
CKH + AMX	1560/2500	781/157	0.5/0.06	0.56	No interaction
CKD + AMX	1560/2500	781/157	0.5/0.06	0.56	No interaction
CKE + AMX	780/2500	1560/157	2/0.06	2.06	No interaction
CKW + AMX	1560/2500	1560/157	1/0.06	1.06	No interaction
CKH + CEF	1560/63	49/4	0.03/0.06	0.09	Synergistic
CKD + CEF	1560/63	3/4	0.002/0.06	0.062	Synergistic
CKE + CEF	780/63	49/4	0.06/0.06	0.12	Synergistic
CKW + CEF	1560/63	6/7.8	0.004/0.12	0.12	Synergistic
CKH + CHL	1560/63	390/8	0.25/0.13	0.38	Synergistic
CKD + CHL	1560/63	781/15.63	0.5/0.25	0.75	No interaction
CKE + CHL	780/63	1560/7.8	2/0.12	2.12	No interaction
CKW + CHL	1560/63	781/31.25	0.5/0.49	0.99	No interaction
CKH + PEN	1560/2500	12/5000	0.01/2	2.01	No interaction
CKD + PEN	1560/2500	12/5000	0.02/2	2.02	No interaction
CKE + PEN	780/2500	1560/156	2/0.06	2.06	No interaction
CKW + PEN	1560/2500	12/5000	0.01/2	2.01	No interaction
MNH + AMP	1560/2500	49/2500	0.03/1	1.03	No interaction
MND + AMP	1560/2500	48.75/2500	0.03/1	1.03	No interaction
MNH + AMX	1560/2500	1560/313	1/0.13	1.13	No interaction
MND + AMX	1560/2500	6/2500	0.004/1	1	No interaction
MNH + CEF	1560/63	6/8	0.004/0.13	0.13	Synergistic
MND + CEF	1560/63	6/8	0.004/0.13	0.13	Synergistic
MNH + CHL	1560/63	24/7.8	0.02/0.12	0.14	Synergistic
MND + CHL	1560/63	390/8	0.25/0.13	0.38	Synergistic
MNH + PEN	1560/2500	12/5000	0.01/2	2.01	No interaction
MND + EN	1560/2500	12/5000	0.01/2	2.01	No interaction

*Values represent triplicates of two independent experiments. CEH, Hexane fraction of Combretum edwardsii; CED, Dichloromethane fraction of Combretum edwardsii; CEE, Ethyl acetate fraction of Combretum edwardsii; CKH, Hexane fraction of Combretum krausii; CKD, Dichloromethane fraction of Combretum krausii; CKE, Ethyl acetate fraction of Combretum krausii; CKW, Water fraction of Combretum krausii; MNH, Hexane fraction of Maytenus nemorosa; MND, Dichloromethane fraction of Maytenus nemorosa*.

The most susceptible bacterial strain used in the study was *S. aureus*. There were 35 synergistic effects out of 44 combinations against this strain (Table 4). Combinations with all antibiotics, except cefotaxime, displayed synergistic activity. The best combinations were water fraction of *C. krausii* and penicillin (FICI = 0.04); ethyl acetate fraction of *C. krausii* and penicillin (FICI = 0.05), hexane fraction of *C. edwardsii* and amoxicillin (FICI = 0.05), hexane fraction of *C. edwardsii* and penicillin (FICI = 0.06), hexane fraction of *C. krausii* and amoxicillin (FICI = 0.06), and ethyl acetate of *C. krausii* and amoxicillin (0.06). In the best combinations, the MICs of penicillin and amoxicillin were reduced from 39 to 1.2 μg/ml and 39 to 2 μg/ml, respectively.

**Table 4:** **Combined effects of different fractions of *Combretum edwardsii, Combretum krausii*, and *Maytenus nemorosa* with antibiotics against penicillin resistant *S. aureus***.

**Combinations**	**Individual MIC (μg/ml)**	**Combination MIC (μg/ml)**	**Individual FIC**	**FIC index (FICI)**	**Interpretation**
CEH + AMP	390/39	1.5/4.9	0.004/0.13	0.13	Synergistic
CED + AMP	195/39	1.5/4.9	0.01/0.13	0.14	Synergistic
CEE + AMP	708/39	12.2/19.5	0.02/0.25	0.27	Synergistic
CEH + AMX	396/39	1.5/2	0.004/0.05	0.05	Synergistic
CED + AMX	195/39	3.1/2.4	0.02/0.06	0.08	Synergistic
CEE + AMX	780/39	49/4.9	0.06/0.13	0.19	Synergistic
CEH + CEF	390/1	195/1	0.5/1	1.5	No interaction
CED + CEF	195/1	195/0.5	1/0.5	1.5	No interaction
CEE + CEF	780/1	780/0.1	1/0.1	1.1	No interaction
CEH + CHL	390/16	98/4	0.25/0.25	0.5	Synergistic
CED + CHL	195/16	24.5/4	0.12/0.25	0.37	Synergistic
CEE + CHL	780/16	12.2/8	0.02/0.5	0.52	No interaction
CEH + PEN	390/39	2/2	0.01/0.05	0.06	Synergistic
CED + PEN	195/39	6/2.4	0.03/0.06	0.09	Synergistic
CEE + PEN	780/39	24/1.2	0.03/0.08	0.11	Synergistic
CKH + AMP	390/39	1.5/4.9	0.004/0.13	0.13	Synergistic
CKD + AMP	390/39	97.7/9.8	0.25/0.25	0.5	Synergistic
CKE + AMP	390/39	1.5/4.9	0.004/0.13	0.13	Synergistic
CKW + AMP	1560/39	1.5/4.9	0.001/0.13	0.13	Synergistic
CKH + AMX	390/39	1.5/2.4	0.004/0.06	0.06	Synergistic
CKD + AMX	390/39	97.7/9.8	0.25/0.25	0.5	Synergistic
CKE + AMX	390/39	1.5/2.4	0.004/0.06	0.06	Synergistic
CKW + AMX	1560/39	97.5/4.9	0.06/0.13	0.19	Synergistic
CKH + CEF	390/1	195/1	0.5/1	1.5	No interaction
CKD + CEF	390/1	195/0.25	0.5/0.25	0.75	No interaction
CKE + CEF	390/1	97.70.1	0.25/0.13	0.38	Synergistic
CKW + CEF	1560/1	195/0.1	0.13/0.13	0.26	Synergistic
CKH + CHL	390/16	1.5/8	0.004/0.5	0.5	Synergistic
CKD + CHL	390/16	98/4	0.25/0.25	0.5	Synergistic
CKE + CHL	390/16	97.5/2	0.25/0.13	0.38	Synergistic
CKW + CHL	1560/16	98/4	0.06/0.25	0.31	Synergistic
CKH + PEN	390/39	6/2.4	0.02/0.06	0.08	Synergistic
CKD + PEN	390/39	1.5/2.4	0.004/0.06	0.06	Synergistic
CKE + PEN	390/39	12/0.6	0.03/0.02	0.05	Synergistic
CKW + PEN	1560/39	12/1.2	0.01/0.03	0.04	Synergistic
MNH + AMP	37/39	1.5/4.9	0.04/0.13	0.17	Synergistic
MND + AMP	49/39	18.5/1.2	0.38/0.03	0.41	Synergistic
MNE + AMP	98/39	0.4/4.9	0.004/0.13	0.13	Synergistic
MNH + AMX	37/39	1.16/10	0.03/0.25	0.28	Synergistic
MND + AMX	49/39	18.5/1.2	0.38/0.03	0.41	Synergistic
MNE + AMX	98/39	0.4/2.4	0.004/0.06	0.06	Synergistic
MNH + CEF	37/1	18.5/0.13	0.5/0.13	0.63	No interaction
MND + CEF	49/1	97.5/2	1.99/2	3.99	No interaction
MNE + CEF	98/1	195/1	1.99/1	2.99	No interaction

*Values represent triplicates of two independent experiments.CEH: Hexane fraction of Combretum edwardsii; CED, Dichloromethane fraction of Combretum edwardsii; CEE, Ethyl acetate fraction of Combretum edwardsii; CKH, Hexane fraction of Combretum krausii; CKD, Dichloromethane fraction of Combretum krausii; CKE, Ethyl acetate fraction of Combretum krausii; CKW, Water fraction of Combretum krausii; MNH, Hexane fraction of Maytenus nemorosa; MND, Dichloromethane fraction of Maytenus nemorosa; MNE, Ethyl acetate fraction of Maytenus nemorosa*.

Considering the number and medicinal importance of the members of the genus *Combretum*, only a few reports exist on their interaction with antibiotics with regards to antibacterial activity. The leaf extract of *C. albidum* potentiated the antibacterial activity of ceftriaxone against multidrug-resistant *Pseudomonas aeruginosa* (Sahu et al., 2014). In a separate study, the leaf extract of *C. molle* also enhanced the antibacterial activities of kanamycin and streptomycin against Gram-negative bacteria including multidrug-resistant strains (Fankam et al., 2015).

## Conclusions

It is important to note how some of the extracts in the present study enhanced the activities of some of the clinically ineffective antibiotics against the two multi-drug Gram-negative bacteria. Examples are the reduction of the MICs of ampicillin and amoxicillin by different fractions of *C. edwardsii* and *C. krausii*. The enhancement could be through the inhibition of beta-lactamases activity or increase in the permeability of the antibiotics thereby making the antibiotics more effective. In other words, they tend to reverse the antimicrobial resistance. The constituents of these plant extracts therefore, have the potential to enhance and restore the activities of some clinically used antibiotics. Since combinations confirmed that *in vitro* effects may not be the same *in vivo*, further studies are still required, especially extensive *in vivo* studies and research on the toxicity of these combinations. As far as we know, this is the first report on the antibacterial activity of *M. nemorosa*, individually and in combination with antibiotics. We intend to follow up this study with bioactivity guided isolation of the bioactive compounds.

## Author contributions

JCC undertook the research and wrote the paper. JvS read and approved the paper.

## Funding

Funding was provided by UKZN in the form of a Postdoctoral Fellowship.

### Conflict of interest statement

The authors declare that the research was conducted in the absence of any commercial or financial relationships that could be construed as a potential conflict of interest.
